# Predicting the axillary lymph node tumor burden in breast cancer patients using ultrasonic radiomics nomogram model

**DOI:** 10.1371/journal.pone.0333172

**Published:** 2025-10-07

**Authors:** Pan Tang, Qi Zhang, Ling-cui Meng, Miao Chen, Sai-Feng He, Jian-Xing Zhang

**Affiliations:** Department of Ultrasound, The Second Affiliated Hospital of Guangzhou University of Chinese Medicine, Guangzhou, Guangdong, China; University of Pisa, ITALY

## Abstract

**Background:**

Assessing axillary lymph node (ALN) tumor burden (low burden: < 3 positive ALNs; high burden: ≥ 3 positive ALNs) preoperatively is essential for guiding treatment strategies. This study aimed to develop a radiomics-based nomogram by integrating clinical data, serologic markers, ultrasound imaging features, and ultrasound-derived radiomics features to predict axillary lymph node metastatic burden in breast cancer.

**Methods:**

A study was conducted on 234 breast cancer patients. Univariate and multivariate logistic regression analyses were used to identify independent risk factors from ultrasound imaging and clinical pathology, constructing a clinical model. Radiomics features were extracted from ultrasound images, and the best features were selected using the Least Absolute Shrinkage and Selection Operator (LASSO) algorithm to construct the Radiomics score. The Radiomics nomogram model was built by combining the Radiomics score and independent risk factors from the clinical model. The performance of the clinical model, radiomics model, and combined model in predicting axillary lymph node tumor burden was evaluated. Model performance was assessed by discrimination, calibration curves, and decision curves.

**Results:**

Results showed that US-reported ALN status and CA153 were independent risk factors for high ALN tumor burden. The radiomics nomogram demonstrated good calibration and discrimination, with an area under the ROC curve of 0.815 (95% CI, 0.755–0.876) for the training set and 0.808 (95% CI, 0.678–0.938) for the testing set. Furthermore, compared to the clinical model and radiomics model, The differences in AUC between the nomogram model and the clinical model, as well as between the nomogram model and the radiomics model, were not statistically significant (nomogram model vs. clinical model: *P* = 0.2078; nomogram model vs. radiomics model: *P* = 0.4161). But the nomogram model provided greater net benefit for all patients in the probability threshold range of 0.05–0.70.

**Conclusions:**

This study highlights the potential of an ultrasound-based radiomics nomogram as a robust and non-invasive predictive tool for evaluating ALN tumor burden, offering valuable guidance for personalized treatment planning in breast cancer.

## Introduction

Breast cancer ranks as the most frequently diagnosed malignancy among women and remains a significant contributor to cancer-related mortality worldwide [1]. Axillary lymph node involvement, occurring in roughly 30–40% of cases, plays a pivotal role in determining clinical staging, guiding treatment decisions, and evaluating prognostic outcomes [2–4]. The ACOSOG Z0011 clinical trial demonstrated that axillary lymph node dissection (ALND) is warranted primarily in patients with three or more positive ALN, promoting a more tailored surgical approach [5]. Subsequently, the assessment of ALN status has shifted from whether metastasis occurs to the degree of ALN tumor burden. Patients with low ALN tumor burden can avoid ALND, which not only does not affect disease-free survival and overall survival but also reduces the occurrence of postoperative complications [4,5]. Patients with high ALN tumor burden benefit from axillary surgery or neoadjuvant chemotherapy, avoiding sentinel lymph node biopsy and saving time and costs [5]. Therefore, accurate preoperative assessment of ALN tumor burden is essential for treatment decision-making.

Ultrasound imaging, characterized by its non-invasiveness and real-time visualization, remains a primary tool for preoperative ALN status evaluation. Nevertheless, its diagnostic accuracy is often influenced by operator expertise and variability in morphological interpretation, necessitating advanced methodologies to improve reliability and reduce subjectivity. Ahmed et al. [6] pointed out that approximately 43.2% of patients with positive axillary ultrasound are low tumor burden patients. This means that over half of the patients with positive axillary ultrasound can avoid ALND, suggesting potential overtreatment based solely on axillary ultrasound assessment [7]. In the era of precision medicine, there is an urgent need for a more effective and personalized approach to address this issue.

Radiomics is an advanced analytical approach that extracts high-dimensional quantitative features from medical images, enabling a detailed exploration of tumor heterogeneity. This methodology facilitates a multifaceted evaluation of imaging attributes, yielding deeper insights compared to traditional single-feature analyses [8–11].

This study aims to establish a radiomics nomogram model based on ultrasound images, integrating clinical and pathological features, serologic markers, ultrasound imaging features, and ultrasonic radiomics features for predicting ALN tumor burden in early-stage breast cancer [12].

## Materials and methods

### Patients

This retrospective study analyzed 234 breast cancer patients who underwent surgical treatment at our hospital between January 1, 2022 and December 31, 2023. Data collection and analysis were completed from January 3 to February 28, 2025. The study was approved by the Medical Ethics Committee of our institution (Ethics Approval Number: ZE2025−002), and being a retrospective study, it was exempt from the requirement for informed consent from the patients. Patients were randomly divided into a training group and a testing group in an 8:2 ratio.

Inclusion criteria: 1. Female patients with primary breast cancer at the initial diagnosis; 2. Patients who had not received adjuvant radiation therapy, chemotherapy, or hormone therapy; 3. Patients who underwent breast-conserving surgery or total mastectomy, confirmed pathologically to have breast cancer; 4. Patients who underwent axillary lymph node dissection and pathological examination; 5. Complete imaging examination data.

Exclusion criteria: 1. Patients who received radiation therapy, chemotherapy, or hormone therapy; 2. Patients with multiple lesions; 3. Patients who had undergone breast augmentation surgery; 4. Patients in pregnancy or lactation; 5. Patients with a history of breast surgery.

### Image acquisition

In this study, the GE LOGIQ E9 color Doppler ultrasound diagnostic instrument with an ML6–15 linear array probe, operating at a frequency of 5–13 MHz, was used. The ultrasound examinations were performed by two attending physicians, Miao Chen and Sai-feng He. Both physicians were blinded to the patients’ clinical data and medical history prior to the examinations. In cases of disagreement between the two, consultation was sought from the chief physician, Jian-Xing Zhang. The final conclusions were made after a consensus was reached among the three physicians. Prior to the examination, all patients did not require any special preparation. Patients were assisted in adjusting their position to a supine position, raising both arms to fully expose both breasts and axillae. Starting from the nipple as the center, a sector scan of the entire breast was performed 2–3 times to determine the location, size, shape, aspect ratio, internal echo characteristics, and presence of microcalcifications within the tumor. Subsequently, color Doppler ultrasound was superimposed to assess the distribution of blood flow within the mass. Each examination also included scanning of the axillary lymph nodes on both sides. All videos and images were stored in the ultrasound device for later analysis.

### Image segmentation and feature extraction

**Fig 1:**
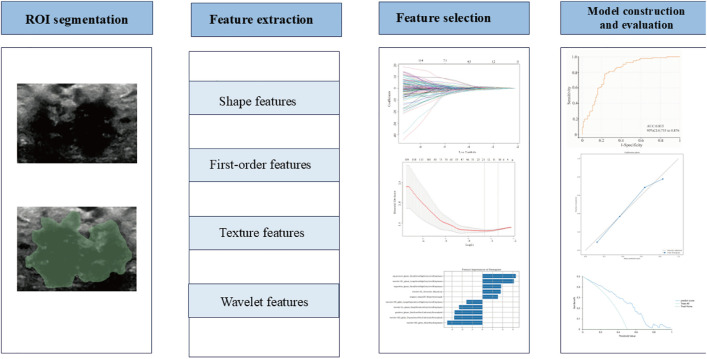
The workflow of this study. This study includes image segmentation, feature extraction, selection of significant radiomic features, model assembly, and performance evaluation.

The region of interest (ROI) was manually delineated by an experienced ultrasound physician with five years of expertise in breast ultrasound examination and diagnosis. The delineation process was performed using the SEG3D2 software (https://www.sci.utah.edu/cibc-software/seg3d.html), focusing on the precise delineation of tumor boundaries on two-dimensional ultrasound images. First-order features, wavelet features, texture features, etc., were automatically extracted from the ROI using pyradiomics (https://pyradiomics.readthedocs.io/en/latest/index.html). All extracted features adhered to the standards set by the Image Biomarkers Standardization Initiative (IBSI). Fig 1 illustrates the workflow of extracting ultrasound radiomics features.

### Feature selection, model establishment, and performance validation

Univariate and multivariate logistic regression analyses were used to identify clinical independent risk factors, and these risk factors were used to construct a clinical prediction model.

Variance threshold, Least Absolute Shrinkage and Selection Operator (LASSO) were employed to reduce the feature dimensions and eliminate irrelevant features to determine the optimal features for constructing Radiomics score (Radscore) and building a radiomics model. Fig 1 illustrates the process of feature selection and model construction of ultrasound radiomics features.

Using clinical independent risk factors and optimal radiomic features to construct a Radscore for building a nomogram model, namely the combined model.

Receiver Operating Characteristic (ROC) curves were utilized to assess the performance of each model. Quantitative analysis was performed by calculating the area under the curve (AUC), accuracy, sensitivity, specificity, and their corresponding 95% confidence intervals (CI). Additionally, Decision Curve Analysis (DCA) and calibration curves were employed to assess the clinical utility of each model and the consistency between actual and predicted probabilities.

### Statistical analysis

Statistical analysis of the data was performed using R software (version 4.1.2, https://www.r-project.org/) and GraphPad Prism 9.0 software. Continuous data were expressed as mean ± standard deviation, and categorical data were presented as frequencies. The chi-square test was used for comparing categorical variables, while the t-test or Mann-Whitney U test was used for comparing continuous variables to assess the consistency of factors in the training and validation sets. The reported levels of statistical significance were two-tailed, and a p-value less than 0.05 was considered statistically significant. R software was utilized for building and evaluating ultrasound radiomics scores and predictive models.

## Results

### Patient characteristics

A total of 234 patients were included in this study and randomly divided into a training set (n = 192) and a testing set (n = 42). Table 1 presents the characteristics of breast cancer patients in the training and testing sets. In the training and testing sets, 96 (50%) and 18 (42.8%) patients, respectively, had a high ALN tumor burden in early-stage breast cancer. There were no significant differences in characteristics between the groups (p > 0.05).

**Table 1:** Characteristics of breast cancer in training and testing sets.

Characteristics	Training set(n = 192)	Testing set(n = 42)	*P*-value
**Age, mean(years)**	56.09 ± 11.06	57.71 ± 11.01	0.3887
**ALN tumor burden**			0.4015
High burden	96	18	
Low burden	96	24	
**Tumor location**			0.6621
Left	94	19	
Right	98	23	
**Tumor quadrant**			0.0543
Up inside	19	3	
down inside	11	7	
down outside	27	9	
up outside	135	23	
**Diameter(mm)**	32.35 ± 20.92	29.76 ± 12.82	0.4414
**Distance from body surface**	6.276 ± 2.996	5.881 ± 2.340	0.0521
**Distance from nipple**	19.07 ± 17.75	21.90 ± 18.40	0.3521
**Margin**			0.9732
not smooth	178	39	
Smooth	14	3	
**burr**			0.5935
Yes	158	36	
No	34	6	
**Shape**			0.7109
irregular	189	41	
regular	3	1	
**aspect ratio**			0.6852
≥1	61	12	
<1	131	30	
**Microcalcification**			0.3606
Yes	162	33	
No	30	9	
**Rear echo attenuation**			0.8961
Yes	130	28	
No	62	14	
**Hyperechoic halo**			0.8991
Yes	53	12	
No	139	30	
**CDFI**			0.1986
0	3	3	
I	30	6	
II	57	10	
III	102	23	
**US BI-RADS**			0.6072
4B	12	1	
4C	51	12	
5	129	29	
**US-reported ALN status**			0.0993
Positive	143	26	
Negative	49	16	
**Mammography BI-RADS**			0.8747
4A	2	0	
4B	1	0	
4C	56	13	
5	133	29	
**CEA (ng/mL)**	4.484 ± 8.588	3,106 ± 3.557	0.3090
**CA153 (U/mL)**	23.57 ± 52.08	19.36 ± 13.00	0.6034
**Pathological microcalcification**			08067
Yes	100	21	
No	92	21	
**T**			0.6770
0	2	0	
1	57	13	
2	113	27	
3	16	1	
4	4	1	
**Histological grading**			0.5255
I	4	0	
II	104	21	
III	84	21	
**Vascular invasion**			0.1630
Yes	114	20	
No	78	22	
**ER**			0.6476
Positive	161	34	
Negative	31	8	
**PR**			0.3096
Positive	130	25	
Negative	62	17	
**HER-2**			0.1854
Positive	66	10	
Negative	126	32	
**Ki-67**			0.1820
Positive	116	30	
Negative	76	12	
**Marginal invasion**			0.2200
Yes	6	3	
No	186	39	

Abbreviations: ALN: Axillary Lymph Nodes; US: Ultrasound; BI-RADS, breast imaging-reporting, and data system; CEA: Carcinoembryonic Antigen; CA: Carbohydrate Antigen; ER: Estrogen Receptor; PR: Progesterone Receptor; HER-2: Human Epidermal Growth Factor Receptor 2; Ki-67: Tumor proliferating cell nuclear antigen 67.

### Clinical model construction and evaluation

**Fig 2:**
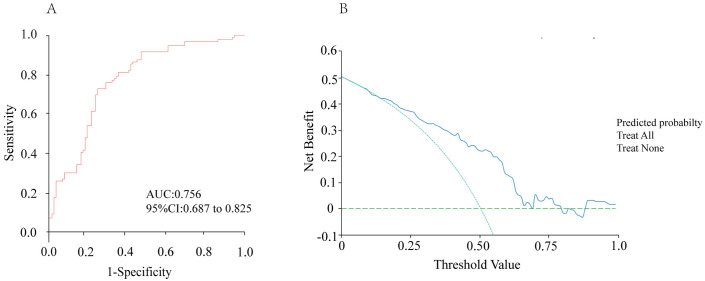
Evaluation of the clinical model. (A) ROC curve of the clinical model; (B) DCA curve of the clinical model.

Table 2 presents the ultrasound and clinicopathological features of patients in the training set. 50% of the patients were in the high ALN tumor burden group, and 50% were in the low ALN tumor burden group. Univariate analysis results showed significant differences between the two groups in US BI-RADS, Mammography BI-RADS, US-reported ALN status, CEA, CA153, Vascular invasion, and Pathological microcalcification (p < 0.05) (Table 2). Multivariate logistic regression analysis identified US-reported ALN status, CA153, and Vascular invasion as independent risk factors (p < 0.05) (Table 3). Considering that the aim of our study was to predict axillary lymph node tumor burden preoperatively, Vascular invasion was not included in the model. The clinical model constructed with US-reported ALN status and CA153 had an AUC of 0.756, sensitivity of 0.718, specificity of 0.738, and 95% CI of 0.687–0.825 (Table 4. Fig 2A). DCA curve analysis assessed the clinical value of the clinical model. The clinical model achieved the maximum net benefit if the threshold probability ranged from 0.15 to 0.69 (Fig 2B).

**Table 2:** Results of univariable analysis in the training set.

Characteristics	OR	95% CI	*P*-value
**Age, mean(years)**	1.023	0.997-1.051	0.088
**Tumor location**	1.182	0.671-2.086	0.565
**Tumor quadrant**	1.338	0.996-1.830	0.059
**Diameter(mm)**	1.014	0.999-1.033	0.107
**Distance from body surface**	1.018	0.926-1.120	0.717
**Distance from nipple**	0.991	0.975-1.007	0.296
**Margin**	0.375	0.099-1.163	0.107
**burr**	1.788	0.846-3.901	0.134
**Shape**	0.332	0.016-2.602	0.340
**aspect ratio**	1.704	0.925-3.177	0.090
**Microcalcification**	1.171	0.536-2.585	0.691
**Rear echo attenuation**	0.909	0.495-1.666	0.758
**Hyperechoic halo**	1.443	0.765-2.748	0.259
**CDFI**	1.141	0.799-1.638	0.468
**US BI-RADS**	1.873	1.151-3.138	0.014
**US-reported ALN status**	6.905	3.235-16.19	<0.0001
**Mammography BI-RADS**	2.355	1.332-4.369	0.005
**CEA (ng/mL)**	1.066	1.012-1.150	0.026
**CA153 (U/mL)**	1.042	1.017-1.071	0.002
**Pathological microcalcification**	2.547	1.431-4.593	0.002
**T**	1.405	0.925-0.217	0.117
**Histological grading**	0.929	0.546-1.581	0.787
**Vascular invasion**	2.640	1.466-4.830	0.001
**Marginal invasion**	0.522	0.821-1.010	0.135
**ER**	0.679	0.306-1.468	0.327
**PR**	0.628	0.341-1.142	0.130
**HER-2**	1.022	0.870-1.218	0.784
**Ki-67**	1.299	0.728-2.328	0.376

Abbreviations: ALN: Axillary Lymph Nodes; US: Ultrasound; CEA: Carcinoembryonic Antigen; CA: Carbohydrate Antigen; ER: Estrogen Receptor; PR: Progesterone Receptor; HER-2: Human Epidermal Growth Factor Receptor 2; Ki-67: Kiel-67; OR: odds ratio; CI, confidence interval.

**Table 3:** Results of multivariate logistic regression analysis in the training set.

Characteristics	OR	95% CI	*P*-value
**US-reported ALN status**	5.291	2.219-13.81	0.000
**CA153 (U/mL)**	1.038	1.010-1.070	0.012
**CEA (ng/mL)**	1.017	0.967-1.093	0.593
**US BI-RADS**	0.850	0.441-1.613	0.620
**Mammography BI-RADS**	1.790	0.853-3.945	0.136
**Pathological microcalcification**	1.625	0.826-3.198	0.159
**Vascular invasion**	2.657	1.349-5.357	0.005

Abbreviations: ALN: Axillary Lymph Nodes; US: Ultrasound; CEA: Carcinoembryonic Antigen; CA: Carbohydrate Antigen; ER: Estrogen Receptor; PR: Progesterone Receptor; HER-2: Human Epidermal Growth Factor Receptor 2; Ki-67: Kiel-67; OR: odds ratio; CI, confidence interval.

**Table 4:** Comparison of diagnostic performance among the clinical model, radiomics model, and nomogram model.

Model	AUC (95%CI)	Sensitivity	Specificity	*P*
**clinical model**	0.756(0.687 to 0.825)	0.718	0.738	0.2078^*^
**radiomics model**	0.778(0.713 to 0.844)	0.719	0.740	0.4161^#^
**nomogram model**	0.815(0.755 to 0.876)	0.781	0.771	

Abbreviations: AUC: Area Under the Curve; CI, confidence interval. “*” represents the p-value for the comparison between the clinical model and the nomogram model, while “#” represents the p-value for the comparison between the radiomics model and the nomogram model.

### Radiomics feature selection and radiomics score establishment

**Fig 3:**
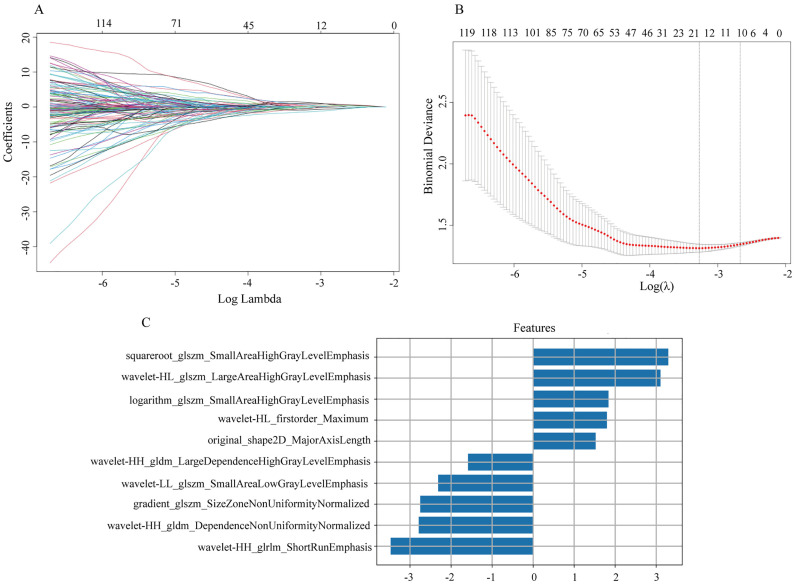
Radiomics features selection using LASSO regression and tenfold cross-test. (A) LASSO coefficient profiles were plotted against the lambda values. (B) Ten radiomics features with non-zero coefficients were obtained through tenfold cross-test. (C) Ranked the 10 key features based on their importance.


A total of 939 ultrasound radiomics features were extracted from each ROI of the primary tumor. Utilizing variance threshold, LASSO, 10 optimal radiomics features were ultimately selected (Fig 3). Subsequently, we obtained the final formula for the RadScore:

RadScore = −3.473*wavelet−HH_glrlm_ShotRunEmphasis

  +3.296*squareroot_glszm_SmallAreaHighGrayLevelEmphasis

    +3.108*wavelet−HL_glszm_LargeAreaHighGraylevelEmphasis

        −2.788*wavelet−HH_gldm_DependenceNonUniformityNormalized

−2.753*gradient_glszm_SizeZoneNonUniformityNomalized

     −2.313*wavelet−LL_glszm_SmallAreaLowGraylLevelEmphasis

  +1.840*logarithm_glszm_SmallArealHighGrayLevelEmphasis

+1.801*wavelet−HL_firstorder_Maximum

     −1.586*wavelet−HH_gldm_LargeDependenceHighGrayLevelEmphasis

         +1.526*original_shape2D_MajorAxisLength−0.487

**Fig 4:**
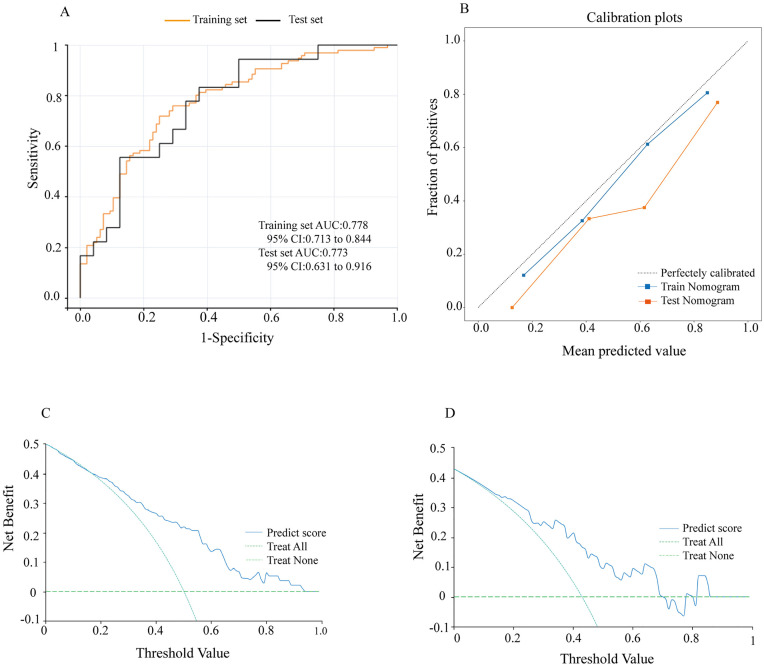
Evaluation of the radiomics model. (A) ROC curves of the radiomics model for the training and testing sets. (B) Calibration curves of the radiomics model for the training and testing sets. (C) DCA curve of the radiomics model for the training set. (D) DCA curve of the radiomics model for the testing set.

A radiomics model was established. In the training set, the radiomics model had an AUC of 0.778 with a 95% CI of 0.713–0.844, while in the testing set, these values were 0.773 and 0.631–0.916, respectively (Fig 4A). The calibration curves of the radiomics model in the training and testing sets demonstrated accurate consistency between the prediction of axillary lymph node tumor burden and pathological validation (Fig 4B). DCA curve were used to evaluate the clinical value of the radiomics model in the training and testing sets. In the training set, the radiomics model achieved the maximum net benefit if the threshold probability ranged from 0.18 to 0.94 (Figs 4C and D).

### Development and clinical application value of the nomogram (Combined Model)

**Fig 5:**
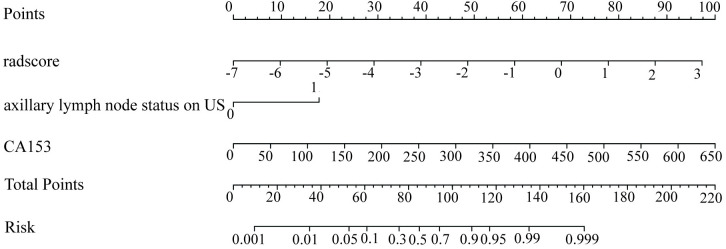
Nomogram predicting the probability of high ALN tumor burden occurrence in breast cancer patients. The nomogram was developed in the training set, including radscore, ALN status on US, and CA153.

**Fig 6:**
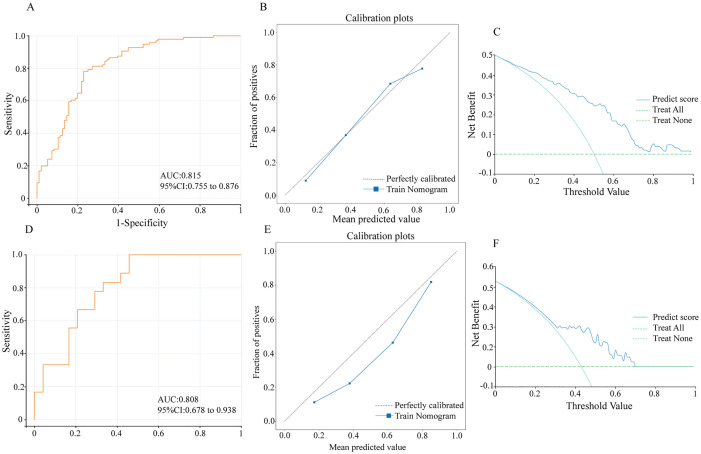
Evaluation of the nomogram model in the training and testing sets. (A) ROC curve of the nomogram model in the training set; (B) Calibration curve of the nomogram model in the training set; (C) DCA curve of the nomogram model in the training set; (D) ROC curve of the nomogram model in the testing set; (E) Calibration curve of the nomogram model in the testing set; (F) DCA curve of the nomogram model in the testing set.

**Fig 7:**
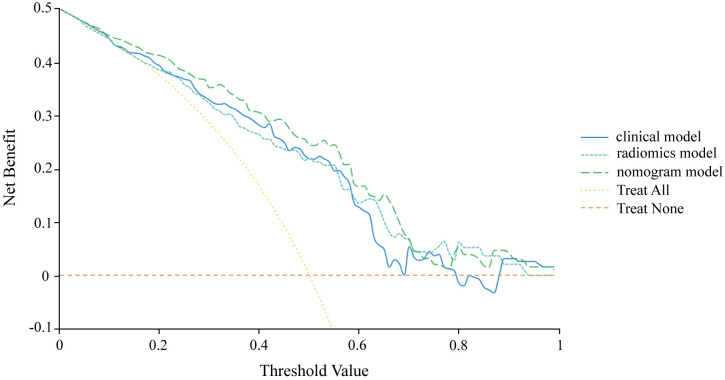
Decision curve analysis illustrating the clinical benefits of the model across various decision thresholds. Curves closer to the top-right corner reflect superior model performance.

A nomogram model, the combined model, was developed by integrating clinical independent risk factors with the radscore to predict ALN tumor burden (Fig 5). The model had an AUC of 0.815 with a 95% CI of 0.755–0.876 in the training set, and in the testing set, these values were 0.808 and 0.678–0.938, respectively (Table 4, Figs 6A and D). The calibration curves of the combined model in the training and testing sets demonstrated accurate consistency between the prediction of axillary lymph node tumor burden and pathological validation (Figs 6B and E). DCA curve analysis was used to evaluate the clinical value of the combined model in the training and testing sets. In the training set, the nomogram model achieved the maximum net benefit if the threshold probability ranged from 0.1 to 1 (Figs 6C and F). Furthermore, compared to the clinical model and radiomics model, The differences in AUC between the nomogram model and the clinical model, as well as between the nomogram model and the radiomics model, were not statistically significant (nomogram model vs. clinical model: *P* = 0.2078; nomogram model vs. radiomics model: *P* = 0.4161, Table 4). But the nomogram model provided greater net benefit for all patients in the probability threshold range of 0.05–0.70 (Fig 7).

### Example of the clinical-radiomics nomogram

Consider a patient diagnosed with a radscore of 2, a positive ALN status on US, and a CA153 level of 150 U/mL. The associated scores were allocated as follows: 88 points for the rad-score of 2, 18 points for the positive ALN status on US, and 22 points for the CA153 level of 150 U/mL. With a cumulative score of 128 points, the predicted risk of high axillary lymph node metastatic burden was 97%, suggesting the need for further diagnostic evaluations or clinical interventions.

## Discussion

For breast cancer patients with preoperative clinical suspicion of ALN metastasis, the traditional treatment strategy typically involves using sentinel lymph node biopsy (SLNB) to assess metastatic status. If SLNB results are positive, further axillary lymph node dissection (ALND) may be performed. However, ALND can lead to complications such as lymphedema, infection, restricted shoulder movement, and vascular or nerve damage [13,14]. Studies have shown that approximately 40% of patients with positive sentinel lymph nodes do not benefit from subsequent ALND [15]. Based on the Z0011 trial results, the American Society of Clinical Oncology recommends that ALND may be avoided in patients with 1–2 positive sentinel lymph nodes without affecting disease-free survival or overall survival [5]. Therefore, the focus of ALN assessment has shifted from confirming metastasis to evaluating tumor burden (low burden: < 3 positive ALNs; high burden: ≥ 3 positive ALNs). The SOUND trial further supports avoiding ALND in patients with low ALN tumor burden by utilizing breast-conserving surgery combined with radiotherapy [16]. Thus, accurate preoperative non-invasive assessment of ALN tumor burden is essential for personalized treatment decision-making.

Although ultrasound is widely used for preoperative ALN evaluation, its diagnostic results are highly influenced by the subjective factors of examiners, resulting in significant inter-observer variability. Additionally, the overlapping morphological features of inflammatory hyperplastic lymph nodes and metastatic lymph nodes make it difficult to differentiate between the two [17]. The reported sensitivity and specificity of ultrasound in predicting ALN tumor burden are only 66% and 73%, respectively, which are unsatisfactory for clinical practice [18]. In this study, we found that 39.4% of patients with ALN-positive results on ultrasound actually had low tumor burden, consistent with Ahmed et al.’s findings (43.2%) [6]. This suggests that relying solely on axillary ultrasound may lead to overtreatment. Through univariate and multivariate logistic regression analyses, we identified ALN ultrasound status and CA153 as independent risk factors for high ALN tumor burden. The clinical model we developed achieved an AUC of 0.756, with a sensitivity of 71.8% and a specificity of 73.8%.

Tumor cells rapidly spread to multiple lymph nodes through lymphatic vessels, making axillary ultrasound a vital tool for evaluating ALN tumor burden [18–20]. Patients with suspicious metastatic lymph nodes on ultrasound are significantly more likely to be diagnosed with high tumor burden, consistent with findings by Shao et al. [21]. CA153, a tumor marker, is closely associated with breast cancer progression and ALN tumor burden [22–24]. It enhances tumor invasiveness through mechanisms such as epithelial-mesenchymal transition (EMT), immune evasion, and lymphangiogenesis [25–28]. Additionally, elevated CA153 levels are often associated with more aggressive tumor phenotypes [28]. This study demonstrated that combining axillary ultrasound with CA153 levels provides a more accurate prediction of ALN tumor burden, offering a reliable basis for patient risk stratification.

Radiomics improves the objectivity of image interpretation by extracting high-dimensional features. In this study, we screened 10 key features out of 939 radiomic features to construct the radiomics model, achieving an AUC of 0.778, with sensitivity and specificity of 71.9% and 74.0%, respectively, outperforming the clinical model. The nomogram model combining radiomics scores and clinical features achieved an AUC of 0.815, sensitivity of 78.1%, and specificity of 77.1%. These results consistent with recent studies [21,29–32]. Although the differences in AUC among the clinical model, radiomics model, and nomogram model did not reach statistical significance (**P* *> 0.05), the nomogram model demonstrated improved numerical performance. Additionally, decision curve analysis (DCA) showed that the nomogram model provided greater net benefit for all patients within the probability threshold range of 0.05 to 0.70. This further validates its clinical utility and indicates that it outperforms the assessment of ALN status by experienced ultrasound physicians based on ultrasound reports.

This study has several limitations. First, its retrospective design may introduce sample bias. Second, the study was conducted at a single center, lacking external validation. Third, we only extracted radiomic features from within breast tumors and did not include features from the surrounding tissues or ALNs. Chen et al. [29] demonstrated that combining radiomic features from both tumors and ALNs improves predictive performance. Future multicenter studies should incorporate radiomic features from tumors, surrounding tissues, and ALNs to further optimize the model’s performance.

### Ethical statement

The authors are accountable for all aspects of the work in ensuring that questions related to the accuracy or integrity of any part of the work are appropriately investigated and resolved. This study was conducted in accordance with the Declaration of Helsinki (as revised in 2013). The medical ethics committee of the Second Affiliated Hospital of Guangzhou University of Chinese Medicine approved the study and waived the requirement for written informed consent due to the retrospective nature of the study.
